# SpineCor vs rigid brace for Adolescent Idiopathic Scoliosis: end of growth results from a retrospective controlled study

**DOI:** 10.1186/1748-7161-8-S2-O56

**Published:** 2013-09-18

**Authors:** Fabio Zaina, Fabio Digiacomo, Sabrina Donzelli, Michele Romano, Alessandra Negrini, Monia Lusini, Salvatore Minnella, Stefano Negrini

**Affiliations:** 1 ISICO Italian Scientific Spine Institute, Milan, Italy

## Background

SpineCor and rigid braces both have both results testifying to their effectiveness in Adolescent Idiopathic Scoliosis (AIS) treatment: an RCT recently compared the two, showing the superiority of rigid braces. In a previous study, we found similar short- term results in curves between 20° and 30° Cobb. 

## Purpose

The objective of this study was to compare the short-term results of the Spinecor vs SPoRT brace for AIS in this selected population. 

## Methods

Study design: retrospective controlled study. Population: Rigid Brace Groups (RBGs) 20 patients (16 female), age 13±1, Cobb 24±5°, ATR 8±3°, TRACE score 7, Risser 0-3. Spinecor Group (SG): 41 patients (33 females) age 13±1, Cobb angle 24±5°, ATR 8±3°, TRACE score 6, Risser 0-3. Both groups were treated with a full-time brace (18 to 23 hours per day upon initial prescription). Clinical and radiological evaluations were performed at the beginning and end of treatment. Main outcome measures: Cobb angle (changes > ±5), ATR, TRACE (changes ≥3). Statistics: Chi square, t-test. 

## Results

Considering patients with more than 5° of Cobb angle change, 40% improved, 45% remained stable and 15% worsened in the RBG vs. 22%, 34% and 44% (p<0.05), respectively. No differences were found for ATR. For TRACE, there were no differences among groups: in RBG, 50% improved and 50% were stable, vs. 65%, 31% and 5% worsened (p>0.05). 

## Conclusions and discussion

Both treatments showed to be effective in improving the aesthetics in AIS. For the other parameters, the SPoRT brace seemed to be more effective than the SpineCor to avoid curve progression, since the number of worsened patients was much higher for the SG. The main limits of the study were the retrospective design and the small population, so further studies are required. 
